# Enhancing college English writing through self-efficacy-based instruction

**DOI:** 10.3389/fpsyg.2025.1668324

**Published:** 2025-09-24

**Authors:** Huiling Jiang

**Affiliations:** School of Applied Foreign Languages, Zhejiang Yuexiu University, Shaoxing, Zhejiang, China

**Keywords:** college English writing, differentiated instruction, self-efficacy, self-regulated learning, writing performance

## Abstract

This quasi-experimental study implemented a 15-week instructional intervention targeting first-year non-English major college students. The intervention integrated differentiated instruction, multi-source feedback, and a structured three-phase writing instruction, emphasizing offering level-appropriate scaffolding, facilitating mastery experiences, and promoting self-regulated learning behaviors. Pre- and post-intervention tests were conducted to examine changes in students’ writing self-efficacy and writing performance. The results indicated significant improvements in both writing confidence and actual writing quality. Key findings are summarized as follows: (1) Students exhibited a significant increase in writing self-efficacy and writing performance, supporting the effectiveness of teaching intervention. (2) A strong positive correlation was observed between writing self-efficacy and writing performance, reinforcing the theoretical proposition that efficacy beliefs positively influence writing engagement and performance. (3) Self-regulated learning behaviors functioned as a critical mediating factor between psychological beliefs and writing performance, highlighting the essential role of agency in the writing process. (4) Differentiated instruction effectively accommodated individual differences in language proficiency and learning needs, enabling learners at all levels to have targeted support and achieve meaningful progress. The study demonstrates that an integrated approach combining psychological development and skill-based training fosters a profound cycle between confidence and competence. This “confidence-skill synergy” model offers a practical and scalable framework for improving English as a Foreign Language writing instruction across diverse classroom contexts.

## Introduction

Writing proficiency serves as a critical indicator for assessing college students’ comprehensive English competence. However, current instruction faces multiple challenges: insufficient allocated class hours limit students’ writing practice opportunities (Fang, 2012); Course instruction demonstrates a tendency to overemphasize teaching delivery and assignment distribution, and disregards teachers’ feedback and students’ comprehension, which leaves students’ writing difficulties unresolved (Yan and Ou, 2021); it is not rare that papers with vague content, unclear logic, or deficient creativity (Jin, 2021) pile in front of teachers. These problems collectively compromise writing quality and undermine students’ confidence in writing. Thus exploring effective methodologies to tap into students’ potential and enhance instructional efficacy has emerged as a significant subject for academic study.

Self-efficacy, introduced by Bandura’s (1997) as a central construct in social cognitive theory, denotes an individual’s beliefs in his capabilities to execute actions required to achieve specific task outcomes. Self-efficacy directly influences academic performance by shaping learners’ goal-setting patterns, sustained effort investment, and resilience when confronting obstacles (Pajares, 2003). Learners with high-self-efficacy demonstrat superior analytical, inferential, and evaluative abilities (Wang, 2021). They are more likely to engage in challenging tasks and strategically deploy metacognitive strategies to optimize learning trajectories (Schunk and DiBenedetto, 2020). In writing contexts, students possessing robust self-efficacy would like to proactively experiment with complex syntactic structures and rhetorical devices, whereas those with lower self-efficacy may shy away from creative expression for fear of errors (Zhou, 2018). This disparity in psychological mechanisms underscores the imperative of integrating self-efficacy principles into pedagogical design.

Metacognitive-strategy instruction effectively enhanced students’ writing self-efficacy, enabling them to confidently assess and modulate their writing processes (Dong and Zhan, 2020). By teaching students how to conceptualize, guiding them through the creative process, and encouraging them to showcase their work, this “three-stage integrated writing teaching model” not only enhances students’ writing abilities but also strengthens their sense of control and accomplishment in tackling writing tasks (Wang, 2021). Feedback approach has likewise been demonstrated as effective in elevating self-efficacy. This method facilitated collaborative learning among students, and provided expanded access to writing guidance and support—all contributing to enhanced writing quality (Cui et al., 2019). Furthermore, web resource-integrated reading-to-writing pedagogy proved effective in augmenting writing self-efficacy. Through digital resource mediation, students achieved deeper task comprehension, leading to measurable improvements in written output (Qiu, 2021).

Taking the aforementioned teaching methods into comprehensive consideration, this study aimed to investigate the application of self-efficacy theory in university English writing instruction from the teacher’s perspective, utilizing student participants from one of the colleges in Zhejiang Yuexiu University as the subjects.

## Methodology

### Subjects

The 20 participants in this study were first-year students majoring in International Trade at one of the colleges in Zhejiang Yuexiu University of Foreign Languages. These students were specially selected by the college from all freshmen and grouped into a single class because they intended to pursue postgraduate studies/study abroad in the future. Based on their National College Entrance Examination (Gaokao) English, converted into a 100-point scale, I divided them into four groups. Those in the 80–89 scoring band comprised 5 students (Group 1), while the 70–79 band included 9 students (Group 2) and the 60–69 band had 4 students (Group 3). Additionally, there were 2 students (Group 4) admitted through separate enrollment examinations—that is, they lacked systematic English instruction due to unavailability of English courses during their secondary vocational education phase.

### Research instruments

This quasi-experimental study employed two locally adapted self-report scales to measure students’ self-efficacy: the English Writing Self-Efficacy Scale (EWSES) and the Learning Behavior Self-Efficacy Scale (LBSES). Both scales were revised to align with the context of college English writing instruction in China, based on established theoretical frameworks, and validated through item analysis, exploratory (EFA), and confirmatory factor analysis (CFA).

The EWSES, adapted from Tang and Xu (2011) (original: 18 items, *α* = 0.90), underwent content review by three English teaching experts and two psychometric specialists for contextual relevance. Following a pilot test (*N* = 50), items with low discrimination (<0.3) or factor loadings (<0.4) were removed, resulting in a 9-item scale focusing on core writing competencies and task-specific coping abilities. EFA supported a unidimensional structure (KMO = 0.812, *χ^2^* = 45.67, *p* < 0.001; 68.4% variance explained; loadings = 0.685–0.814). CFA confirmed good model fit (*χ^2^/df* = 2.15, *RMSEA* = 0.056, *CFI* = 0.94, *TLI* = 0.92). The revised scale demonstrated good reliability (*α* = 0.78) and validity (AVE = 0.62, CR = 0.85).

The LBSES, adapted from Liang (2000) and grounded in Printrich and De Groot’s (1990) framework (original: 10 items, *α* = 0.817), was refined through consultation with five experienced English teachers to emphasize “knowledge mastery” and “model text analysis.” Generic learning behavior items were removed, and five new items capturing strategic and reflective writing behaviors were added, yielding a 10-item version. EFA indicated unidimensionality (KMO = 0.834, *χ^2^* = 412.33, *p* < 0.001; 65.2% variance explained; loadings = 0.681–0.783). CFA confirmed acceptable fit (*χ^2^*/*df* = 2.08, *RMSEA* = 0.059, *CFI* = 0.93, *TLI* = 0.91). The revised scale showed good internal consistency (*α* = 0.82).

Both scales used a 5-point Likert format (1 = strongly disagree, 5 = strongly agree). With clear items and sound psychometric properties, they are suitable for measuring writing self-efficacy and its behavioral underpinnings in this study.

### Research procedure

#### Questionnaire administration and writing assessments

The teaching intervention was implemented in three progressive stages: foundational development, genre exploration, and intensive training (see Table 1). A pre-test was administered in Week 1, consisting of the English Writing Self-Efficacy (EWSE) questionnaire and a timed writing task (prompt: Go to college abroad or at home). The results were used to assess students’ initial proficiency and psychological state, thereby informing the setting of instructional goals, the design of tiered content, and pedagogical planning. A post-test was conducted in Week 14 (1 week before the College English Test Band four—CET-4), including EWSE and Learning Behavior Self-Efficacy (LBSE) questionnaires, along with a CET-4 simulated writing task (prompt: Live in a big city or a town). The two writing tasks were comparable, as both aligned with the CET-4 in topic type, cognitive demand, genre characteristics, and scoring criteria. This longitudinal design systematically examined the impact of the 15-week intervention on students’ self-efficacy and writing proficiency, and provided empirical evidence for exploring the dynamic relationship between psychological beliefs and academic performance.

**Table 1:** Compatibility plan for teaching process and differentiated instruction strategies.

Phases	Week(s)	Common content	Differentiated methods (by group)	Support	Feedback focus
Foundation building	1	Pre-test (writing and self-efficacy)	Collective engagement	–	–
	2–3	Common essay structures	G1: Analyze the logical structure of high-scoring model essays G2: Complete standard structure writing G3: Use template-filling writing G4: Gradually transit from sentence to paragraph construction	G1: Extended reading materials G2: Writing checklists G3: Sentence pattern templates G4: One-on-one tutoring, visual prompts	G1: Developmental, heuristic feedback G2: Structural completeness, conjunction use G3: Grammatical correctness, basic conjunctions G: Subject-verb agreement, tense, sentence completeness (corrective + encouraging)
	4	CET-4 writing requirements and conventions	Collective engagement	–	–
Genre exploration	5–6	Comparative & contrast essay (subject-by-subject, point-by-point)	G1: Use complex conjunctions (e.g., whereas, conversely) G2: Use basic comparison structures (e.g., similarly, however) G3: Sentence pattern scaffolding (e.g., One difference is…/Another similarity is…) G4: Paired sentence sorting → paragraph writing.	Same as above	Same as above
	7–8	Cause-effect essays	G1: Analyze underlying causes of social phenomena G2: Write about personal cause-effect experiences G3: Use picture prompts for simple sentences G4: Complete “cause → effect” matching exercises.	Same as above	Same as above
	9–10	Argumentative essays (support-consent, problem-solution)	G1: Write full argumentative essays with multi-angle reasoning G2: Write essays with 2 supporting arguments G3: Complete reason selection & sorting tasks G4: Orally express opinions (e.g., “I think… because…”)	Same as above	Same as above
Final push	11–12	Multi-genre on the same topic	G1: Timed simulations, focus on linguistic diversity G2: Improve structural completeness G3: Strengthen grammar and conjunction use G4: Correct fundamental errors (S-V agreement, tense).	Same as above	Same as above
	13	Review common errors and high-scoring model essays	Collective engagement	–	–
	14	Post-test (writing and self-efficacy)	Collective engagement	–	–
	15	In-depth self-feedback on work	Collective engagement	–	–

G1–G4: student groups based on initial proficiency. Support and feedback for Groups 1–4 in phases 2–3, 5–6, 7–8, 9–10, and 11–12 follow the same strategies as specified in weeks 2–3.

### Instructional design

Guided by Dewey’s (1938) pragmatic theory of education and informed by pre-test results, this study implemented a 15-week instructional intervention aimed at helping students master foundational English writing skills, become familiar with common CET-4 essay types and patterns, avoid frequent errors, and improve their writing performance (WP). The instruction was organized into three progressive phases: foundational development, genre exploration, and intensive training (see Table 1). A differentiated instruction model—"uniform pacing with tiered support”—was adopted: all students participated synchronously in lectures and model text analyses and completed weekly writing tasks of the same genre, ensuring systematic input of core knowledge. Meanwhile, based on students’ language proficiency and pre-test performance, adjustments were made in learning objectives, task complexity, and support strategies to enable individualized instruction (see Table 1). The feedback system integrated technological and human elements: students first received automated feedback via the online platform^1^ to self-correct errors; teachers then used the tool Wenxiaoyan (AI Writing Assistant) to provide personalized written feedback, focusing on essay structure (introduction–body–conclusion) and content quality (unity, support, coherence). Tailored emphasis was applied for different student groups (see Table 1), creating a multidimensional interaction that addressed cognitive, behavioral, emotional, and motivational aspects, thereby enhancing learning outcomes.

## Results and discussion

### A significant improvement in EWSE

Following the 15-week teaching intervention, students’ overall writing self-efficacy significantly increased (M = 2.75, SD = 0.79) compared to pretest levels (M = 1.56, SD = 0.33), with a highly significant difference, *t*(19) = 8.32, *p* < 0.001. The paired-samples *t*-test yielded a Cohen’s *d* of 1.47, indicating a large effect size and confirming the intervention’s substantial impact (see Table 2).

**Table 2:** Descriptive statistics, paired comparisons, and correlations by group.

Group	Pre-EWSE	Post-EWSE	*p* (Pre vs. Post)	LBSE	*r* (LBSE and Post-EWSE)
	M (SD)	M (SD)		M (SD)	
1	2.02 (0.05)	3.58 (0.18)	< 0.001	3.80 (0.51)	0.59
2	1.58 (0.07)	2.42 (0.65)	0.001	2.63 (1.00)	0.98
3	1.22 (0.13)	2.91 (0.96)	0.013	3.23 (0.74)	0.97
4	1.06 (0.08)	1.83 (0.39)	0.111	2.00 (0.71)	1
All	1.56 (0.33)	2.75 (0.79)	<0.001	2.98 (0.94)	0.94

This finding echoes the core tenet of Bandura’s (1997) self-efficacy theory: individuals’ efficacy beliefs can be reshaped through mastery experiences. In this study, differentiated teaching interventions provided structured opportunities for students to accumulate successful experiences of “I can write,” thereby enhancing their writing self-efficacy (Zimmerman, 2000). Among the four groups, Group 3 demonstrated the largest mean improvement (ΔM = 1.69), reflecting substantial gains from a relatively low starting point (M = 1.22). Group 4 began with the lowest initial self-efficacy (M = 1.06) and exhibited a positive but non-significant increase (ΔM = 0.77, *p* = 0.111 > 0.05). Given the very small sample size in Group 4 (*n* = 2), statistical conclusions are limited; however, the observed improvement suggested that even learners with initially low confidence may respond to targeted support. This aligns with the educational principle that “a low starting point does not equate to low potential” (Ushioda and Dörnyei, 2021), underscoring the value of differentiated instruction in fostering growth across diverse learner profiles. Nevertheless, the generalizability and long-term stability of these gains, particularly in small subgroups, warrant further investigation with larger samples.

However, the post-test mean value of students’ self-efficacy (M = 2.75) still falls below the moderate level proposed in foreign research (M = 3.5; Oxford and Burry-Stock, 1995) and the benchmark for non-English major students in China (M = 3.355; Tang and Xu, 2011). This indicates that while current teaching methods are effective, they have not fully activated students’ efficacy potential. The underlying reasons may be attributed to the following: students’ “perfectionist anxiety” towards writing and their “awe” of high-scoring compositions undermine their foreign language enjoyment (FLE); their “uncertainty” about long-term writing ability development may also weaken their perseverance and grit in sustained effort. The weakening of these two psychological pathways undermines their efficacy beliefs. Future teaching should transcend skill training and systematically incorporate the cultivation of a “growth mindset” (Dweck, 2006), emphasizing the mediating role of key psychological resources such as “grit” and “foreign language enjoyment (FLE)” (Hu et al., 2022), to help students achieve a stable sense of efficacy in believing “I can write well.

### Positive correlation between LBSES and EWSE

The data analysis revealed a highly significant positive correlation between students’ self-efficacy for learning behaviors and their post-test scores in writing self-efficacy (*r* = 0.94, *p* < 0.001; see Table 2). This overall strong correlation underscores the pivotal mediating role of learning behaviors in bridging general self-efficacy beliefs and specific cognitive aspects of writing ability, thereby corroborating Pajares and Johnson’s (1994) assertion that “learning behaviors serve as a critical bridge for translating self-efficacy into actual performance.”

However, significant differences were observed across the groups. Groups 2 and 3 both exhibited very strong and statistically significant positive correlations (*r* = 0.98, *p* < 0.001; *r* = 0.97, *p* = 0.030 < 0.05). This suggested that despite differences in initial academic performance, once students possess strong Self-Regulated Learning (SRL) capabilities, a stable and positive reciprocal relationship can be established between their learning behaviors and writing self-efficacy. This finding is consistent with the results reported by Wang (2023). In contrast, Group 1 showed a moderate positive correlation trend, but it was not statistically significant (*r* = 0.59, *p* > 0.05), which may be attributed to its small sample size (*n* = 5) and limited within-group individual variability. This may be attributed to their relatively fixed learning patterns, resulting in lower behavioral plasticity. This suggests that the effectiveness of SRL interventions may be relatively limited for individuals with high self-efficacy and stable learning behaviors. As for Group 4, the correlation coefficient was 1.00; however, due to the extremely small sample size (*n* = 2), it lacks validity for statistical inference. The data suggested that although students in this group demonstrated strong writing motivation, their self-regulated learning abilities were insufficient, leading to lower levels of self-efficacy. This phenomenon is consistent with the core tenet of SRL theory: motivation must be mediated and transformed through a goal-directed behavioral system to effectively enhance an individual’s self-efficacy.

The aforementioned results not only corroborated Zimmerman’s (2002) model of self-regulated learning but also resonated strongly with recent empirical findings by Wang (2023) on SRL-based writing strategies. They suggest that college English writing instruction should transcend the conventional “content-language” binary framework and systematically integrate the cultivation of self-regulated learning competencies.

### Positive correlation between EWSE and WP

Table 3 shows a significant positive correlation between writing self-efficacy and writing performance (pre-test: *r* = 0.70, *p* < 0.001; post-test: *r* = 0.84, *p* < 0.001), validating Bandura’s (1997) proposition that efficacy beliefs influence task performance. A consistent trend is observed across all groups, with post-test correlation coefficients generally higher than those at pre-test. This indicates that, as the instructional intervention progresses, the alignment between psychological beliefs and actual competence strengthens, and self-efficacy gradually transforms from a “vague expectation” into “verifiable confidence.” This finding resonates with Golparvar and Khafi (2021), who demonstrated that second language writing self-efficacy can effectively predict writing performance. Learners with higher self-efficacy are more likely to actively mobilize strategic resources and engage in the writing process, thereby enhancing the quality of their writing. The marked increase in the correlation between self-efficacy and performance at post-test suggests that students may have progressively developed a more systematic repertoire of writing strategies during the instructional process, thereby reinforcing the positive cycle of “belief—behavior—outcome.”

**Table 3:** Descriptive statistics and correlations between EWSE and WP.

Group	Pre-WP	Pre-EWSE	*r* (Pre)	Post-WP	Post-EWSE	*r* (post)
	M (SD)	M (SD)		M (SD)	M (SD)	
1	69.4 (4.45)	2.02 (0.05)	0.72	81.9 (2.8)	3.58 (0.18)	0.87
2	67.8 (4.68)	1.58 (0.07)	0.75	77.0 (4.0)	2.42 (0.65)	0.85
3	67.5 (2.89)	1.22 (0.13)	0.71	76.8 (3.0)	2.91 (0.96)	0.77
4	50.5 (0.71)	1.06 (0.08)	1	65.0 (7.1)	1.83 (0.39)	1
All	66.4 (6.53)	1.56 (0.33)	0.7	77.6 (6.1)	2.75 (0.79)	0.84

Table 3 shows that the correlation coefficient for Group 4 is 1.00. Although this result, based on an extremely small sample size (*n* = 2), lacks the statistical power for valid inference, qualitative observations suggest the following interpretation: for learners with weak foundational skills, an increase in initial self-efficacy may trigger a “motivational turning point,” prompting a shift from “avoiding writing” to “attempting expression,” thereby leading to a rapid improvement in performance in the short term. This highlights the importance of implementing a “confidence-first” strategy in remedial instruction—psychological empowerment can serve as a critical catalyst for re-engaging learners, even when their linguistic competence remains underdeveloped (Hiver et al., 2022).

In summary, Table 3 not only reveals a strong association between self-efficacy and writing performance but also underscores the underlying psychodynamic mechanism: self-efficacy is not merely an outcome of writing development, but also an intrinsic driver that promotes learning engagement, strategic deployment, and continuous improvement. Therefore, college English writing instruction should achieve an organic integration of “psychological development” and “skill training” to foster a virtuous cycle between the two.

### Significant improvement in students’ writing WP

Following the instructional intervention, there was a significant improvement in writing performance across all students (M = 77.6 vs. 66.4, *p* < 0.001, *d* = 1.47), providing empirical support for the effectiveness of differentiated instruction based on self-efficacy principles in enhancing EFL writing skills. Despite varying initial levels, all groups achieved statistically significant progress (*p* < 0.05, see Table 4).

**Table 4:** Descriptive statistics and paired *t*-test results for WP by group.

Group	Sample Size	Pre-WP	Post-test score	Mean difference	*t*	*p*	*d*
	*n*	M (SD)	M (SD)				
1	5	69.4 (4.1)	81.9 (2.8)	12.5	6.63	0.0029	3.47
2	9	67.8 (3.2)	77.0 (4.0)	9.2	6.44	0.0002	2.52
3	4	67.5 (5.6)	76.8 (3.0)	9.3	4.97	0.0159	2.11
4	2	50.5 (2.1)	65.0 (7.1)	14.5	15.81	0.0407	6.45
All	20	66.2 (8.3)	77.6 (6.1)	11.4	6.55	< 0.001	1.47

The extent of improvement varied among the groups. Group 1 exhibited substantial gain in scores (ΔM = 12.5, *d* = 3.47), which can be attributed to the alignment between their strong language proficiency and advanced cognitive training, aligning with Tomlinson’s (2017) emphasis on providing challenges for advanced learners. Although Groups 2 and 3 had similar pre-test writing scores (67.8 vs. 67.5), they exhibited structural differences in their English proficiency at the time of enrollment. Notably, both groups showed comparable improvements post-intervention (ΔM = 9.2 vs. 9.3), with similar post-test scores (77.0 vs. 76.8). This “compensatory effect” suggests that differentiated instruction effectively activated learning behavior efficacy in Group 2, enabling them to rapidly catch up with the initially higher-performing Group 3. This finding corroborates the existing argument that differentiated instruction, through targeted support, can bridge the gap caused by pre-existing ability differences, thereby promoting both procedural fairness and developmental equity (Mohamed et al., 2025).

Group 4 demonstrated a high average score increase (ΔM = 14.5) in the post-test. However, due to the extremely small sample size (*n* = 2), the stability and generalizability of this result are highly limited. The calculated effect size (*d* = 6.45) is severely distorted by the instability of the denominator (standard deviation), rendering it practically meaningless. This observation indicates that while targeted instructional interventions may yield significant progress for students with weak foundational skills, further validation through studies with larger sample sizes is necessary.

## Conclusion

Teaching interventions centered on enhancing self-efficacy can significantly bolster English writing self-efficacy and actual writing performance among college freshmen. This study verifies that self-efficacy plays a pivotal role in regulating learning behaviors, sustaining learning motivation, and promoting academic achievement. The teaching model demonstrated clear practical value.

Further analysis revealed that self-regulated learning behaviors served as a crucial mediating factor between psychological beliefs and academic outcomes, which corroborates the core tenets of the Causality in Triadic Interaction posited in Bandura’s (1977, 1978, 1986) social cognitive theory. Meanwhile, the integration of differentiated teaching strategies effectively addressed the individual differences among students in terms of language proficiency and learning needs, enabling students at varying levels to make significant progress and contributing to the promotion of educational equity. The combination of psychological support and skill training fostered a virtuous cycle where “confidence enhances writing, and writing boosts confidence.”

Therefore, by systematically cultivating students’ self-efficacy beliefs and providing effective scaffolding for their learning behaviors, writing instruction based on self-efficacy offers an effective pathway for college English education that balances the development of language proficiency with the enhancement of psychological resilience.

### Limitations

This study exhibited several limitations: (1) The research sample was from first-year students of a single institution who were non-English majors and consisted of selected students with “intentions to pursue postgraduate studies/study abroad.” Their learning motivation and foundational proficiency levels may be higher than those of the general student population. Consequently, the external validity of the conclusions can be limited and requires verification among a broader student demographic. (2) The total sample size was relatively small (*N* = 20), particularly with Group 4 having only two participants (*n* = 2), which led to unstable effect size estimates and increased the risk associated with statistical inferences. (3) This study involved a short-term intervention. Although significant improvements in self-efficacy and writing performance were observed, the long-term stability of these changes remains unclear and necessitates further follow-up.

### Future research

Future research can delve deeper in the following directions: (1) Conduct multi-center studies across multiple universities of different types, incorporating a broader range of student populations to enhance the representativeness and generalizability of the research; (2) Extend the intervention and observation periods (e.g., over an academic year or longer) to track the long-term developmental trajectories of students’ self-efficacy and writing abilities, exploring their stability and influencing factors; (3) Integrate technological tools such as AI-powered writing feedback systems and adaptive learning platforms to investigate how to efficiently implement personalized support in large-class settings, reduce teacher workload, and enhance the operability of differentiated instruction; (4) Integrate variables such as growth mindset, foreign language learning enjoyment, and grit to construct a more comprehensive psychological model, revealing their mediating and moderating roles in the development of self-efficacy.

## Data Availability

The original contributions presented in the study are included in the article/supplementary material, further inquiries can be directed to the corresponding author.

## Footnotes

^1^ www.pigai.org

## Appendix

**Table A1:** Factor loading of the EWSES.

Item number and content	Loading values
(1) I possess knowledge of the general principles of English writing.	0.768
(2) I can correctly spell all words used in compositions.	0.743
(3) I can accurately apply grammatical categories of words (e.g., nouns, verbs, adjectives).	0.814
(4) I can express emotions or personal experiences to peers/instructors in English (e.g., surprise, preferences, frustration, complaints, perspectives/opinions on events, aspirations, or ideals).	0.690
(5) I can comprehensively narrate events to peers/instructors in English, including development, resolution, temporal/spatial elements, participants, and causal relationships.	0.723
(6) I can appropriately employ diverse writing techniques in specific writing tasks.	0.685
(7) I can promptly identify the genre based on College English Test Band 4 (CET-4) writing prompts.	0.732
(8) I can efficiently organize writing frameworks in response to CET-4 essay prompts.	0.789
(9) I can compose a 120-180-word passage within 30 min meeting requirements: relevant and complete in content, coherent and logical in meaning, with structure and expression conforming to stylistic conventions.	0.719

**Table A2:** Factor loading of the LBSES.

Item number and content	Loading values
(1) I maintain regular English study habits between the college entrance examination and matriculation.	0.783
(2) I aim to achieve high scores in the CET-4 writing section to enhance my overall test performance.	0.752
(3) I endeavor to comprehend thematic discussions during instructional delivery.	0.724
(4) I systematically document key instructional points and conscientiously review them post-class.	0.681
(5) I proactively engage in classroom questioning and collaborative discourse.	0.736
(6) During preview sessions, I synthesize essential content and analyze writing methodologies from source materials.	0.710
(7) When practicing writing, I deliberately recall acquired compositional knowledge.	0.741
(8) Following each writing exercise, I refine my compositions with evaluative feedback.	0.695
(9) I critically examine model essays to extract effective expressions and writing techniques.	0.703
(10) I consistently reflect on factors impeding my English writing fluency.	0.722
